# Superior mediastinal paraganglioma associated with von Hippel-Lindau syndrome: report of a case

**DOI:** 10.1186/1477-7819-12-74

**Published:** 2014-03-29

**Authors:** Tsuyoshi Takahashi, Hiroshi Nogimura, Ken Kuriki, Ryo Kobayashi

**Affiliations:** 1 Department of Surgery, Yaizu City Hospital, 1000 Dobara, Yaizu-City, Shizuoka 425-8505, Japan; 2 Department of Pathology, Yaizu City Hospital, 1000 Dobara, Yaizu-City, Shizuoka 425-8505, Japan

**Keywords:** Mediastinal paraganglioma, Pheochromocytoma, von Hippel-Lindau syndrome, *VHL* gene

## Abstract

Extra-adrenal pheochromocytomas are termed paragangliomas. Paragangliomas in the mediastinum, especially the superior mediastinum, are extremely rare. It is known that paragangliomas or pheochromocytomas occur in combination with von Hippel-Lindau syndrome. We present the case of a non-functional superior mediastinal paraganglioma in a patient with von Hippel-Lindau syndrome, without a familial history suggestive of the condition. This case highlights that we should be aware of possible sporadic von Hippel-Lindau syndrome in patients with a mediastinal paraganglioma.

## Background

Pheochromocytomas and paragangliomas are neuroendocrine tumors that arise from the sympathetic or parasympathetic paraganglia. Mediastinal paragangliomas are rare and slow-growing tumors. Some patients with pheochromocytomas or paragangliomas show an association with an inherited condition such as von Hippel-Lindau syndrome or multiple endocrine neoplasia type 2. We report the case of a non-functional superior mediastinal paraganglioma in a patient with von Hippel-Lindau syndrome.

## Case presentation

**Figure 1:**
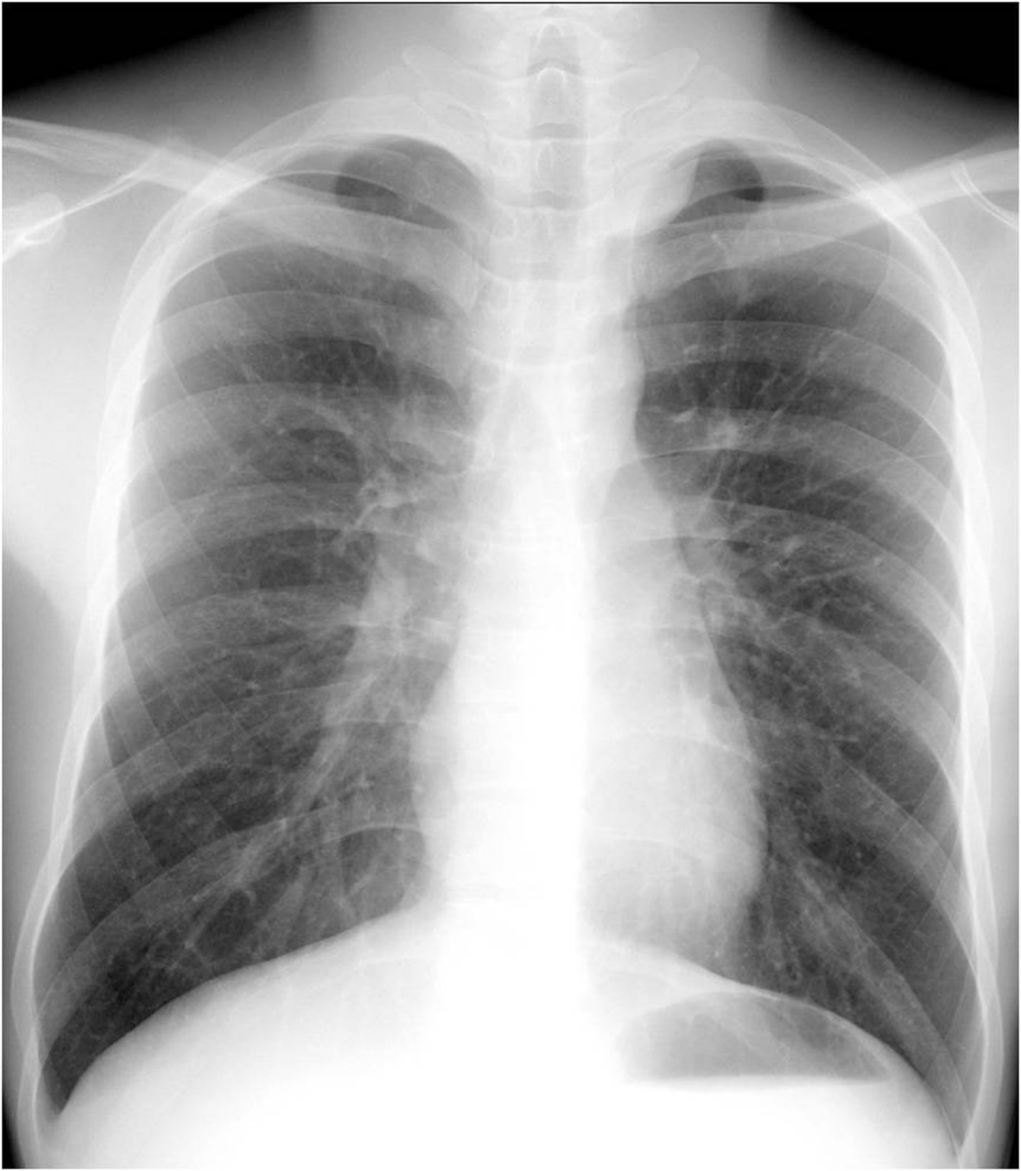
Chest radiograph showing an abnormal shadow in the superior mediastinum.

A previously healthy 18-year-old man was referred to our hospital due to an abnormal shadow on a chest radiograph performed as part of a health check (Figure 1). The patient had no past medical or familial history of note.

**Figure 2:**
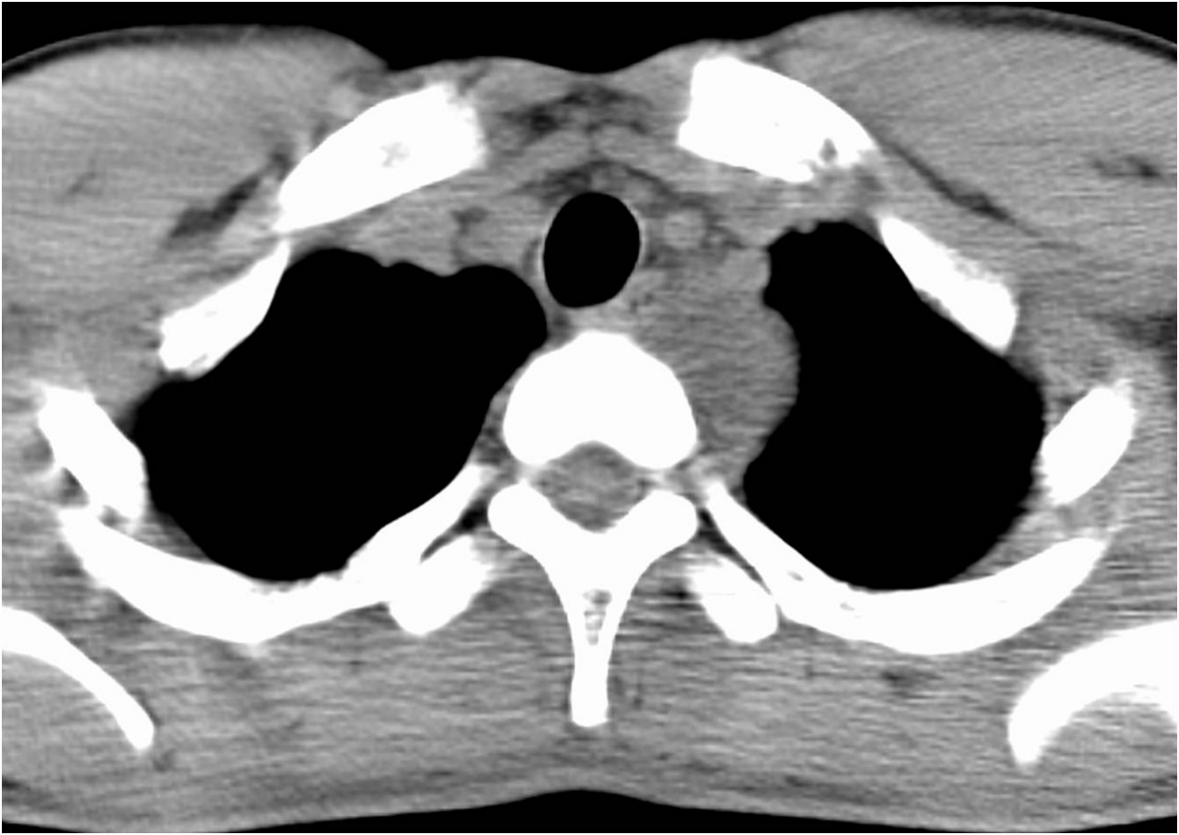
Chest computed tomography showing a 36 × 21 × 30 mm superior mediastinal mass located between the left common carotid and subclavian arteries, trachea, and spine.

There were no abnormalities on physical examination, and laboratory data were within normal limits. Chest computed tomography (CT) without contrast revealed a 36 × 21 × 30 mm superior mediastinal mass located between the left common carotid and subclavian arteries, trachea, and spine (Figure 2).

**Figure 3:**
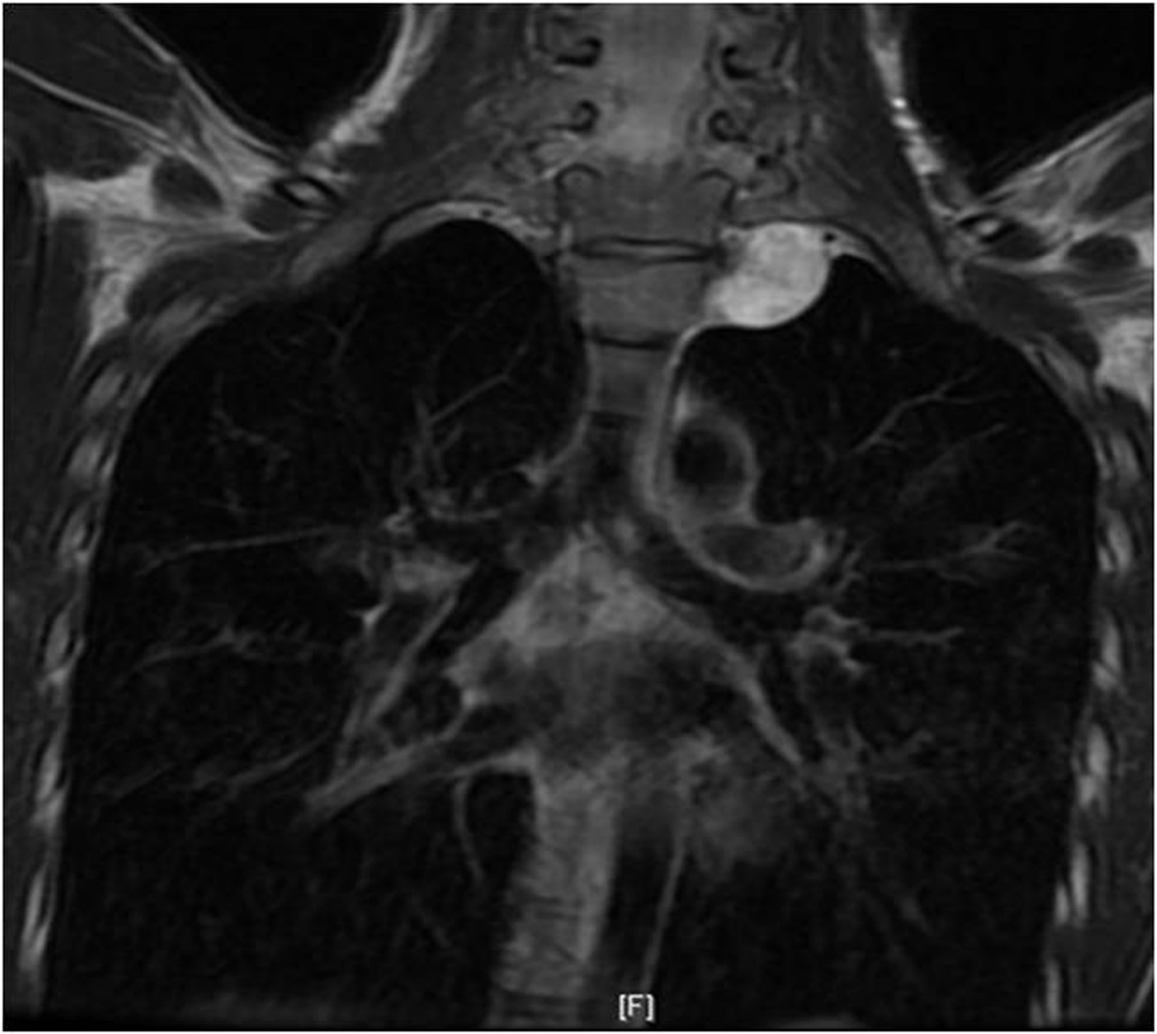
Chest magnetic resonance image (T2-weighted) revealing an inhomogeneous-intensity mass with no direct invasion of the left common carotid and subclavian arteries.

Chest magnetic resonance imaging (MRI) showed an inhomogeneous-intensity mass with no direct invasion of the left common carotid and subclavian arteries (Figure 3). Preoperatively, we diagnosed the mass as a superior mediastinal neurogenic tumor.

**Figure 4:**
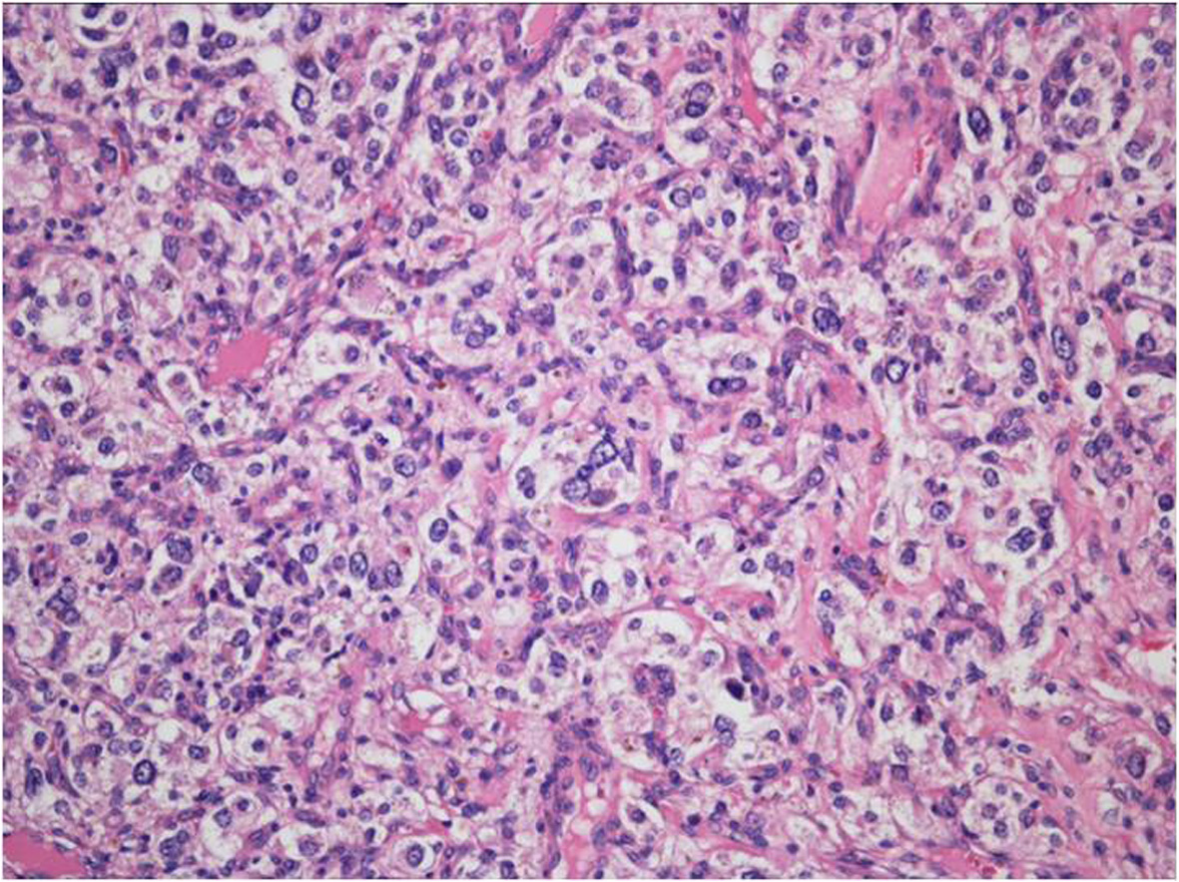
A paraganglioma consisting of cells arranged in nests (‘zellballen’) with a vascular stroma (H & E stain).

**Figure 5:**
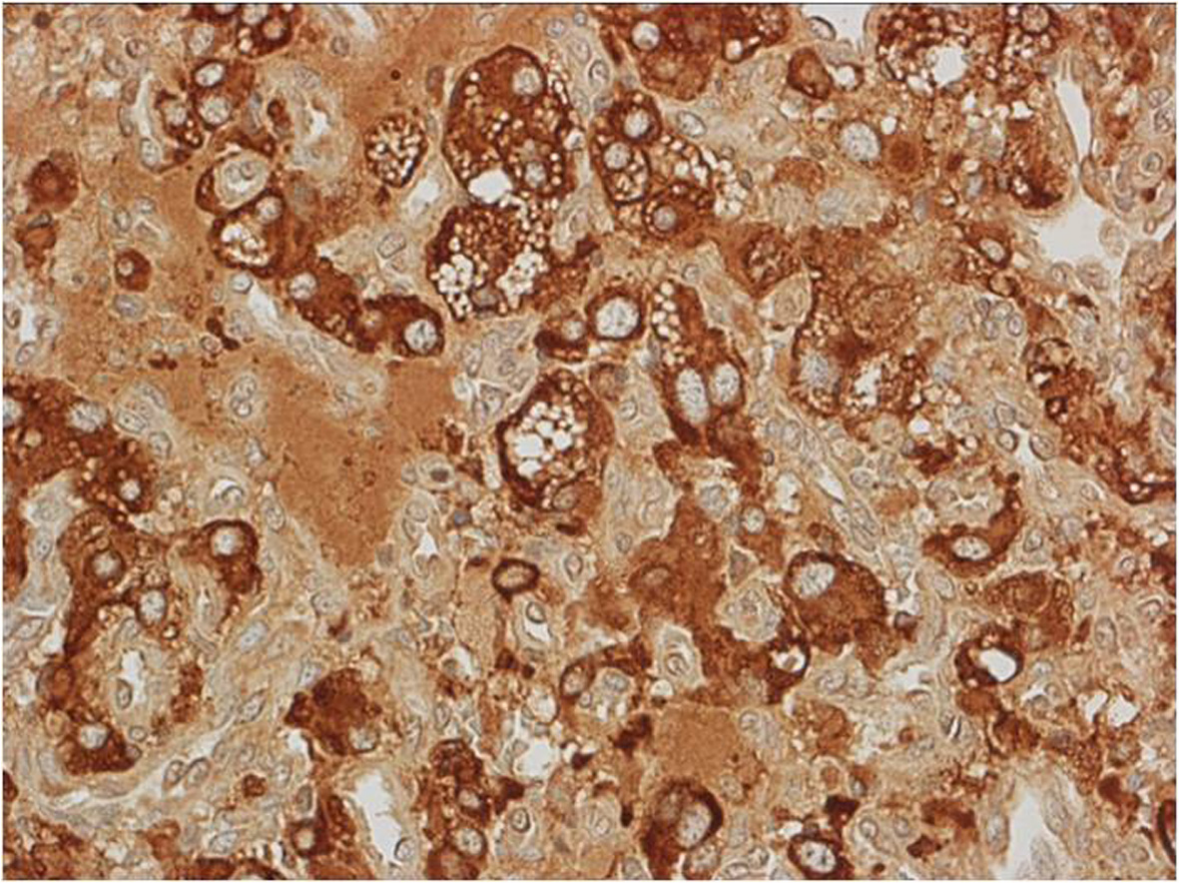
Immunohistochemistry showing a positive immunoreaction for chromogranin A.

Complete resection of the tumor was performed by video-assisted thoracoscopic surgery using a direct approach through a left supra-clavicular incision. No remarkable blood pressure changes and little blood loss occurred during the operative procedure. Histologic findings showed that the tumor consisted of cells arranged in nests (‘zellballen’) with a vascular stroma (Figure 4). Immunohistochemistry showed a positive immunoreaction for chromogranin A (Figure 5) and synaptophysin.

The patient’s postoperative course was uneventful, without Horner’s syndrome. Six months after the first operation, CT for postoperative follow-up revealed a right adrenal gland and pancreatic tumor and para-aortic lymph node enlargement. These tumors were resected, and histopathologic analysis led to the diagnosis of a right pheochromocytoma, para-aortic paraganglioma, and endocrine tumor. Genetic analysis revealed a mutation of the *VHL* gene (exon2 TTT → TGT), indicating von Hippel-Lindau syndrome. Further analyses revealed no abnormalities in other organs associated with von Hippel-Lindau syndrome. There was no sign of recurrence on CT scans six months and three years after the first surgery.

## Discussion

Extra-adrenal paragangliomas usually arise in the abdomen. Paragangliomas in the mediastinum, especially the superior mediastinum, are extremely rare. Functional mediastinal paragangliomas are often discovered during the investigation of unexplained hypertension or other typical symptoms such as palpitations, headaches, or profuse sweating. On the other hand, non-functional mediastinal paragangliomas are asymptomatic and are usually found incidentally during the course of imaging studies for other reasons. The preoperative diagnosis of mediastinal paragangliomas is generally confirmed through elevated plasma and urinary levels of catecholamines and the methylated metabolites metanephrines. The location of the tumor is determined using CT, MRI, or ^131^I or ^123^I metaiodobenzylguanidine scintigraphy. About 5 to 10% of pheochromocytomas and paragangliomas are malignant [1]. However, the diagnosis of malignant paragangliomas is generally difficult. Malignant paragangliomas are usually diagnosed in patients with distant metastasis, commonly found in the lungs, bone, or liver.

Pheochromocytomas and paragangliomas are present in about 25 to 33% of patients with an inherited condition such as von Hippel-Lindau syndrome [1]. von Hippel-Lindau syndrome is an autosomal-dominant disorder, and mutation of one copy of the *VHL* tumor suppressor gene is associated with the development of the tumors. Recently, it has been reported that *de novo* mutations seem to play a greater role in von Hippel-Lindau syndrome than previously thought [2]. In our case, since there was no mutation of the *VHL* gene in the patient’s family, suggestive of von Hippel-Lindau syndrome, we suspect that the patient had a *de novo* mutation of the gene.

The treatment of paragangliomas involves complete tumor removal. In one report, the complete tumor removal rate was 76.9%, and only 20.0% of patients who were thought to have undergone complete tumor resection experienced late recurrence [3]. The survival rate associated with complete resection was 84.6%. On the other hand, the survival rate was only 50.0% for patients who underwent only a biopsy or partial resection and adjuvant treatment (*P* < 0.01) [4]. Thus, complete tumor resection is an important prognostic factor. For functional paragangliomas, to avoid perioperative hypertensive crisis, alpha-adrenergic blockade is often used preoperatively. Beta-adrenergic blockade and calcium channel blockers can also be used for uncontrolled hypertension in conjunction with alpha-adrenergic blockade [3]. When the tumor is hypervascular and invades surrounding vascular structures, complete tumor resection may be challenging and result in massive bleeding. To prevent perioperative massive bleeding, preoperative embolization of the tumor-feeding vessels may be essential [5].

## Conclusion

We report the extremely rare case of a young patient with a superior mediastinal paraganglioma associated with von Hippel-Lindau syndrome, without a familial history suggestive of the condition. We could safely perform complete resection of the superior mediastinal paraganglioma by video-assisted thoracoscopic surgery using a direct approach through a left supra-clavicular incision. This case highlights that we should be aware of possible sporadic von Hippel-Lindau syndrome in patients with multifocal paragangliomas.

## Consent

Written consent was obtained from the patient for the use and publication of this case report and the accompanying images. A copy of the written consent is available for review from the Editor-in-Chief of this journal.

## Abbreviations

CT: computed tomography; MRI: magnetic resonance imaging.

## Competing interests

The authors declare that they have no competing interests.

## Authors’ contributions

TT collected the information, researched the literature, and wrote the article. KK performed the histological examination and helped prepare the manuscript. HN and RK helped in literature research and edited the final version of the manuscript. All authors read and approved the final version of the manuscript.
